# Exercise for Trismus Prevention in Patients with Head and Neck Cancer: A Network Meta-Analysis of Randomized Controlled Trials

**DOI:** 10.3390/healthcare10030442

**Published:** 2022-02-26

**Authors:** Ya-Hui Wang, Yi-Ai Huang, I-Hui Chen, Wen-Hsuan Hou, Yi-No Kang

**Affiliations:** 1 Division of Speech Therapy, Department of Physical Medicine and Rehabilitation, Wan Fang Hospital, Taipei Medical University, Taipei 11696, Taiwan; elainew@w.tmu.edu.tw (Y.-H.W.); 107273@w.tmu.edu.tw (Y.-A.H.); 2 School of Nursing, College of Nursing, Taipei Medical University, Taipei 11031, Taiwan; ichen4@tmu.edu.tw; 3 Master Program in Long-Term Care and School of Gerontology Health Management, College of Nursing, Taipei Medical University, Taipei 11031, Taiwan; 4 Department of Physical Medicine & Rehabilitation and Geriatrics & Gerontology, Taipei Medical University Hospital, Taipei 11031, Taiwan; 5 Center of Evidence-Based Medicine, Department of Education, Taipei Medical University Hospital, Taipei 11031, Taiwan; 6 Cochrane Taiwan, Taipei Medical University, Taipei 11031, Taiwan; 7 Evidence-Based Medicine Center, Wan Fang Hospital, Taipei Medical University, No. 111, Section 3, Xinglong Road, Taipei 11696, Taiwan; 8 Research Center of Big Data and Meta-Analysis, Wan Fang Hospital, Taipei Medical University, Taipei 11696, Taiwan; 9 Institute of Health Policy & Management, College of Public Health, National Taiwan University, Taipei 100, Taiwan; 10 Department of Health Care Management, College of Health Technology, National Taipei University of Nursing Health Sciences, Taipei 112, Taiwan

**Keywords:** mouth opening, trismus, prophylactic, oral cancer, laryngeal cancer, pharyngeal cancer

## Abstract

A common side effect of managing head and neck cancer is trismus, which devastates patients’ quality of life. The purpose of this study was to investigate prophylactic exercise interventions for preventing trismus and difficulty in mouth opening in head and neck cancer. Five databases were searched for randomized controlled trials. Network meta-analysis was performed with risk ratio (RR) or mean difference (MD) with 95% confidence interval (CI). This study finally included 11 randomized controlled trials (*n* = 805). Trismus risk in patients who received exercise with phone call follow up (E + P) was significantly lower than those received usual care (RR = 0.42; 95% CI: 0.29 to 0.61) and exercise alone (RR = 0.33; 95% CI: 0.18 to 6.22). Mouth opening in usual care was significantly lower than in the tri-integrated strategy group (MD = 15.22; 95% CI: 8.88 to 21.56). Exercise is recommended for preserving mouth opening distance in patients with head and neck cancer. Tri-integrated strategies could be an effective method for preventing trismus.

## 1. Introduction

Head and neck cancer is the seventh most common type of cancer worldwide. In 2018, 887,659 new-onset cases occurred, and 453,307 patients died due to head and neck cancers [1]. The majority of patients with head and neck cancers received their diagnosis near age 50 years [2]. Many medical treatments have been implemented to manage head and neck cancers [3,4] and extend life expectancy. The average life expectancy of patients with head and neck cancers is approximately 10 to 13 years [2]. However, some side effects may occur after head and neck cancer treatment, and the effects can be long-term [3,4]. A common complication is trismus, which devastates patients’ quality of life. Trismus refers to difficulty in opening the mouth or in jaw mobility; specifically, a patient has trismus when the distance between their upper and lower central incisors is less than 35 mm when they open their mouth as wide as possible [5,6]. Trismus reduces speech intelligibility and results in poor oral hygiene, extremely poor dental care, weak eating or chewing performance (and even dysphagia), pain when making facial expressions, poor social interaction, reduced quality of life, difficulty with physical examination, and lack of compliance with cancer treatment [7]. Hence, the prevention of trismus is essential for patients with head and neck cancers.

Unfortunately, trismus frequently occurs after common head and neck cancer treatments, including surgery, radiotherapy, chemotherapy, and chemoradiotherapy. These treatments are being used with increasing frequency [3,4], and the treatments often cause edema, muscle fibrosis, trismus, and poor swallowing [8,9]. Approximately 30% to 50% of patients with head and neck cancers had trismus when they were diagnosed, and 31% to 87% of patients developed trismus after they received treatment [8,10,11,12,13]. According to one study, their ability to open their mouths dramatically decreased within the first 9 months following head and neck cancer treatment [11].

Patients with head and neck cancers develop trismus quickly if they do not receive rehabilitation [14], and they should be referred for rehabilitation to prevent or treat trismus as soon as it is diagnosed [4]. The main strategy of trismus prevention and treatment is exercise, including mouth-opening exercises and jaw range of motion exercises [8]. Nevertheless, few syntheses have provided an overview of this topic. To close this gap, this study empirically investigated whether the ability to open the mouth can be preserved and whether trismus is preventable. This study’s research question in patient/intervention/control/outcome/study design (PICOS) format is as follows:

(a) Patient: patients with head and neck cancers;
(b) Intervention: oral/jaw exercise;
(c) Control: usual care;
(d) Outcome: trismus event and mouth opening distance;
(e) Study design: randomized controlled trial.

## 2. Methods

The purpose of the present meta-analysis was to synthesize studies that met the following conditions: randomized controlled trials (RCTs) that recruited patients with head and neck cancer diagnosis, regardless of cancer stage (I to IV), recency of onset, or recurrence. Furthermore, interventions for the prevention of trismus or difficulty in mouth opening must have been implemented before trismus was diagnosed. The study had to include adults (aged ≥ 18 years), but no restrictions on trial location were imposed. Consequently, the exclusion criteria were as follows: (a) nonhuman subjects were examined, (b) the study included candidates with a diagnosis outside of head and neck cancer criteria, (c) cases did not involve mouth opening or trismus, (d) irrelevancy, (e) prevention not the study focus, or (f) not an RCT. Study protocol could be found in PROSPERO (CRD42020179953).

### 2.1. Data Sources and Evidence Selection

The authors used relevant keywords regarding mouth opening, trismus, head and neck cancer, and prevention in both the medical subject heading terms and the free text for literature and searched these terms in the Cochrane Library, CINAHL, EMBASE, PUBMED, and Web of Science databases. No filters based on study design, age, location, publication date, or language were applied to the searches. The comprehensive search was performed in December 2021 (Supplementary Material S1) details the search process. Two authors classified the articles independently using titles and abstracts according to eligibility criteria. Then, they retrieved the full texts of potentially eligible articles and further examined references according to the eligibility criteria. If they had any disagreement on the evidence selection, they made the final decision in a meeting.

### 2.2. Data Extraction and Quality Evaluation

Afterward, the two authors extracted the information and data regarding study design, duration of the study, the number of cases, ages, sexes, treatments, cancer treatment types, trismus incidence, mouth opening distance (mean and standard deviation), and measurements on the timing of outcomes. These data were recorded using Microsoft Excel. Mouth opening distance was defined and measured by the maximal distance between interincisal, central incisor, and alveolar ridges or between superiors and inferiors of frenulum labii in patients without teeth [10,15,16]. Based on the extracted information, the two authors evaluated the risk of bias in each RCT independently, and when their judgments of the risk of bias differed, they engaged in discussion to reach a consensus. In accord with the Cochrane Handbook [17], the two authors independently evaluated the risk of bias in the selected studies’ selection, performance, attrition, detection, and reporting processes. They also made the final decision through discussion if they disagreed on the evaluation.

### 2.3. Data Synthesis and Analysis

Outcomes in the present study were primarily the incidence of trismus and mouth opening distance. According to measurement time points, these outcomes could be classified as short term (≤3 months) and long term (6 to 12 months). Trismus incidence was a binary variable, and risk ratio (RR) was used to present a pooled estimate. Because mouth opening distance was a continuous variable, the mean difference (MD) in mouth opening distance was used for comparisons. For precision and significance tests, this study calculated the 95% CI of RR for trismus incidence and the 95% CI of MD for mouth opening distance.

This synthesis tested heterogeneity among direct evidence and performed *I*^2^ measures. High heterogeneity was defined as *I*^2^ ≥ 50%. The present study pooled direct and indirect evidence using a contrast-based method and assessed inconsistency and small-study effects. Because the present network meta-analysis comprise both two-arm and three-arm trials, inconsistency tests were performed using a design-by-treatment interaction model. Small-study effects were evaluated through the application of an adjusted funnel plot and Egger’s regression intercept test. When all treatments of the present meta-analysis are nonsignificant with large effect sizes raising clinical concerns, *p* values are presented to help readers determine the most optimal treatment for trismus prevention. For example, the treatment with the highest *p* score might be the optimal trismus prevention strategy. All analyses are performed with R in RStudio. For the present meta-analysis, further confidence ratings for trismus incidence and mouth opening distance are performed separately [18], and evidence that may provide clinicians with a rapid, adequate understanding of how to use exercise to prevent trismus in patients with head and neck cancers is summarized.

## 3. Results

**Figure 1:**
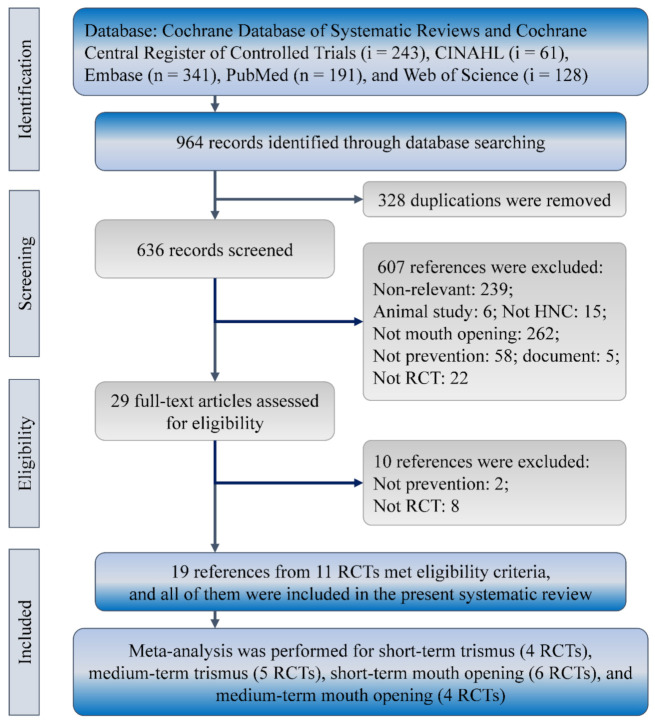
Flowchart of evidence selection for the synthesis of exercise in preventing trismus among patients with head and neck cancer. HNC, head and neck cancers; RCT, randomized controlled trial.

A total of 19 of 964 identified references satisfied the eligibility criteria of the present synthesis (Figure 1). The 19 references reported 11 RCTs from Brazil (*n* = 90), China (*n* = 104), Denmark (*n* = 100), England (*n* = 71), the Netherlands (*n* = 55), Sweden (*n* = 66), Taiwan (*n* = 68), and the United States (*n* = 258) between 2001 and 2018 [8,9,15,16,19,20,21,22,23,24,25,26,27,28,29,30,31,32,33].

### 3.1. Characteristics and Quality of Included Studies

The meta-analysis included 11 RCTs with 805 cases and covering five categories of interventions, including usual care, exercise alone, a combination of exercise and an instrument (e.g., a tongue blade or a device such as TheraBite or Dynasplint), exercise with phone call follow ups, and a tri-integrated intervention strategy (combination of exercise, instrument, and phone call follow up). The term “usual care” refers to education for the patient in terms of cancer treatment, prevention method, nutrition, oral care, and similar information. Available information from the included articles revealed that a total of 581 males and 187 females whose mean ages ranged from 54 to 62.5 years were analyzed. Accessible data revealed oropharynx cancers (*n* = 201), cancer of the oral cavity and floor of the mouth (*n* = 180), and nasopharynx cancers (*n* = 113) were study topics, but cancer type was not specified in some instances. The included RCTs covered major types of head and neck cancers. Approximately 634 patients received radiotherapy for a duration of 5 to 7 weeks, with total doses ranging from 50 to 72.5 Gy; approximately 431 participants received chemotherapy, and 254 patients underwent head and neck cancer surgery. Table 1 presents relevant information about each trial. Most trials seemed to be at high risk of performance bias, detection bias, and attrition bias (Supplementary Table S2), and they consequently contributed to some concerns and major concerns in the confidence ratings of the network meta-analysis of trismus incidence and mouth opening distance (Table 2).

### 3.2. Trismus Incidence

**Figure 2:**
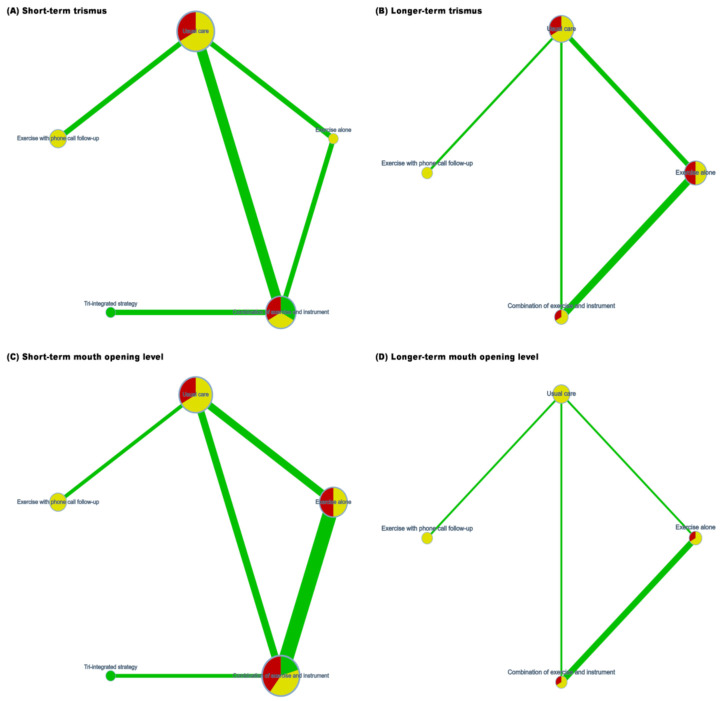
Network geometry of the consistency model for (**A**) short-term trismus, (**B**) longer-term trismus, (**C**) short-term mouth opening level, and (**D**) longer-term mouth opening level.

**Figure 3:**
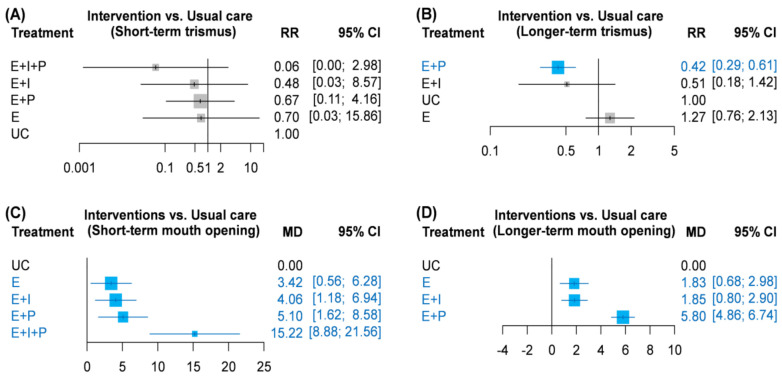
Forest plots of (**A**) short-term trismus, (**B**) longer-term trismus, (**C**) short-term mouth opening level, and (**D**) longer-term mouth opening level. CI, confidence interval; E, exercise alone; E + P, exercise with phone call follow up; E + I, exercise and instrument; E + I + P, exercise and instrument and phone call follow up; MD, mean difference; RR, risk ratio; UC, usual care.

Seven of the included RCTs reported trismus events after randomization [15,16,23,24,27,28,30]. Data on short-term trismus incidence were presented in four RCTs (Figure 2A), and long-term trismus incidence was available in five RCTs (Figure 2B). The consistency model of short-term trismus incidence consisted of five nodes, including usual care, exercise alone, a combination of exercise and an instrument, exercise with phone call follow up, and a tri-integrated strategy. No serious heterogeneity was identified in each pairwise comparison (Supplementary Figure S3). Although the consistency model yielded no significant findings, large effect sizes of the tri-integrated strategy (RR = 0.06; 95% CI: 0.001–2.98) and the combination of exercise and an instrument (RR = 0.48; 95% CI: 0.03–8.57) may be of interest to clinicians (Figure 3A). A similar trend is apparent in the *p* values (Supplementary Figure S4).

Regarding long-term trismus incidence, the four-node consistency model examined usual care, exercise alone, a combination of exercise and an instrument, and exercise with phone call follow ups. The pooled results of direct evidence regarding long-term trismus did not exhibit serious heterogeneity (Supplementary Figure S5). Trismus incidence among patients who received exercise with phone call follow ups was significantly lower than that among patients who received usual care (RR = 0.42; 95% CI: 0.29–0.61) or exercise alone (RR = 0.33; 95% CI: 0.18–6.22; Figure 3B).

### 3.3. Mouth Opening

A total of six RCTs reported sufficient information on mouth opening for quantitative synthesis [16,19,24,27,28,30]. All six RCTs presented treatment outcomes of short-term mouth opening, and four of them reported long-term mouth opening. Regarding short-term mouth opening, network meta-analysis involved usual care, exercise alone, a combination of exercise and an instrument, exercise with phone call follow up, and a tri-integrated strategy. The consistency model had a closed loop and consisted of four trials with a two-arm design and two trials with a three-arm design. Heterogeneity was evident in the direct evidence of pairwise comparisons of usual care and combinations of exercise and instruments (*I*^2^ = 72%; *p* = 0.06), as well as between exercise alone and combinations of exercise and instruments (*I*^2^ = 58%; *p* = 0.07). However, the same effect direction could be observed in all studies comparing usual care and combinations of exercise and instruments (Supplementary Figure S6). Results of the consistency model revealed that exercise alone (MD = 3.42; 95% CI: 0.56–6.28), the combination of exercise and an instrument (MD = 4.06; 95% CI: 1.18–6.94), exercise with phone call follow up (MD = 5.10; 95% CI: 1.62–8.58), and a tri-integrated strategy (MD = 15.22; 95% CI: 8.88–21.56) were associated with significantly higher mouth opening distance than usual care (Figure 3C). Moreover, patients who received a tri-integrated intervention had significantly higher mouth opening distance compared patients who received exercise alone (MD = 11.80; 95% CI: 5.59–18.00), combinations of exercise and an instrument (MD = 11.16; 95% CI: 5.51–16.81), and exercise with phone call follow up (MD = 10.12; 95% CI: 2.89–17.35).

Regarding long-term mouth opening, a four-node consistency model was formed by usual care, exercise alone, combinations of exercise and instrument, and exercise with phone call follow up. Direct evidence of long-term mouth opening ability did not exhibit statistical heterogeneity (Supplementary Figure S7). Mouth opening distance in patients who received exercise alone (MD = 1.83; 95% CI: 0.68–2.98), combinations of exercise and an instrument (MD = 1.85; 95% CI: 0.80–2.90), or exercise with phone call follow up (MD = 5.80; 95% CI: 4.86–6.74) was significantly higher than in those who received usual care (Figure 3D). Furthermore, patients who received exercise with phone call follow up had significantly higher mouth opening distance than did patients who engaged in exercise alone (MD = 3.97; 95% CI: 2.48–5.46) or a combination of exercise and an instrument (MD = 3.95; 95% CI: 2.54–5.36).

### 3.4. Quality of Life

Five of the eligible RCTs tended to investigate quality of life [8,9,15,22,27], while three of them did not report data [9,15,22]. The other two RCTs provided results of quality of life for before–after comparison within each group, and they did not report result for between group comparison. Moreover, they measured quality of life differently. Therefore, the present study did not synthesize their data quantitatively. Based on the available data and information, exercise might improve quality of life [8]. Furthermore, the combination of exercise and instrument appeared to have greater benefits on quality of life than exercise alone among patients with head and neck cancer [27].

### 3.5. Inconsistency Test and Small-Study Effects

Results of the inconsistency tests were nonsignificant in our network meta-analysis of short-term trismus (*Q* = 1.12; *p* > 0.10), long-term trismus (*Q* = 0.93; *p* > 0.10), short-term mouth opening distance (*Q* = 0.93; *p* > 0.10), and long-term mouth opening distance (*Q* = 0.48; *p* > 0.10). According to our funnel plot and the Egger’s test, small-study effects appeared to not seriously affect pooled estimates of short-term trismus (*p* > 0.10; Supplementary Figure S8), long-term trismus (*p* > 0.10; Supplementary Figure S9), and short-term mouth opening distance (*p* > 0.10; Supplementary Figure S10). Although funnel plots of long-term mouth opening distance were asymmetric (*p* < 0.10; Supplementary Figure S11), further analysis of the *p* curve revealed a non-*p*-hacking pattern among the included RCTs (Supplementary Figure S12).

## 4. Discussion

### 4.1. Key Findings

This study found that oral exercise may effectively preserve both short- and long-term mouth opening ability and reduce trismus incidence in patients undergoing treatment for head and neck cancers. Moreover, engaging in exercise combined with an instrument may lead to improved mouth opening distance and reduced trismus incidence, particularly in those patients who regularly receive follow-up phone calls. Notably, the effect size of the tri-integrated strategy on mouth opening distance achieved clinical significance. These findings imply that phone call follow ups by speech therapist are essential to recovering patients because the follow ups provide remote support for adherence to treatment regimens [28].

Patients who adhered to the treatment protocol and daily mouth opening training significantly improved their mouth opening distance from 16 mm to 27 mm [34]. Effective clinical practice occurs when patients and health providers communicate well, and patients ought to play an active role in working with health professionals by adhering to the treatment recommendations [35]. Indeed, adherence to exercise programs plays a crucial role in prevention outcomes [16]. However, the adherence rate dramatically decreased from week 4 onward, and a steep drop-off from 70% to 38% was observed between week 6 and week 12 after cancer-related treatment [36]. The nonadherence may be due to patients having low awareness of their muscle condition [37]. In fact, muscles atrophy in only 3 days if they cannot reach their range of motion during movement [38], and trismus develops most rapidly within the first 6 months after radiotherapy [11]. Moreover, poor adherence is an obstacle to delivering efficient health care, obtaining satisfactory outcomes, achieving recovery, and saving on treatment costs [35,39,40]. Accordingly, promoting adherence to exercise is essential for patients with head and neck cancers, and phone call follow ups might be a practical solution.

Although health education on skills or actions may support behavioral changes that support trismus prevention or management, follow up is still necessary due to differing barriers at each stage of behavioral change [39]. Phone calls by speech therapist are a type of follow up intended to provide remote support [28], and relevant feedback or reminders may be delivered through phone call follow up [24]. Such relevant feedback and social support could promote adherence to exercise regimens [41]. Hence, phone call follow ups are important because they provide feedback with social support, resulting in increases in exercise adherence. Remarkably, social support is positively correlated with health-related quality of life and is crucial for patients with head and neck cancers [42], but patients with head and neck cancers reported that they had received poor social support 1 year after treatment [43]. Phone call follow ups conducted by professionals may meet the requirements of these patients and further promote adherence to exercise regimens. Moreover, health professionals could strengthen adherence through intervention delivery and detect potential nonadherence [39], and the implementation of multimodal interventions is recommended for improving adherence [44].

Moreover, if relevant programs encourage patients to start exercise within 6 months after cancer treatment, such programs may promote patient behavioral changes and improve their adherence [41,45]. Otherwise, trismus may develop within the first 6 months after cancer-related treatment [11]. Our results echoed these perspectives because our results indicated that exercise has more obvious effects on long-term trismus prevention.

### 4.2. Limitations

The present meta-analysis has the following limitations. First, our study identified five strategies for preventing trismus among patients with head and neck cancers, but various cancer sites and stages were mixed together. No of the eligible RCTs reported findings by cancer site or stage, wherefore the present study can only provide overall effects of treatment strategies on head and neck cancers. It would be meaningful to distinguish effects of treatment strategy on different cancer sites in the future. Second, the modalities implemented in the included RCTs’ varied considerably, and subgroup cannot be carried out due to insufficient data for further network meta-analysis. Moreover, more effective interventions, namely the tri-integrated strategy and exercise with phone call follow ups, were only applied in two RCTs, respectively. However, these two treatments relied on a single trial without comparison with exercise alone, and this situation might have decreased the confidence in the evidence even though our synthesis did not observe serious inconsistency between direct and indirect comparisons. Therefore, we recommend that further studies compare exercise alone, exercise with phone call follow up, and tri-integrated strategies. Third, most (91%) of the included RCTs were designed with small sample sizes (*n* < 100). Although our study did not detect a serious small-study effect, the findings may be underpowered. Fourth, some concerns originate from forms of study bias, including performance bias, detection bias, and attrition bias. Practically, exercise intervention is difficult to blind to both medical professionals and participants, and patients with cancer might be lost to follow up in exercise programs due to other major factors, such as discomfort after cancer-related treatments, cancer progression, or mortality. Because the present synthesis considered objective outcomes and because the attrition may not be primarily have been caused by a trial intervention-related adverse event, the biases appeared to be nondifferential. Fifth, most of the studies only reported results within 1 year, and insufficient data were reported for trismus or mouth opening after a 2-year follow up. Because trismus may develop after years of cancer treatment, this topic still warrants more high-quality RCTs with longer follow up in the future.

## 5. Conclusions

Preventive exercise is a necessary intervention for ensuring that patients with head and neck cancers maintain the ability to open their mouths; nonetheless, neither an instrument nor phone call follow up was concurrently applied in the studies we analyzed. Our study results indicate that combining exercise and instruments with phone call follow ups could be an effective treatment approach for preventing trismus among patients with head and neck cancers. Further research on long-term effects (>2 years) with more consistent intervention protocols and study designs is warranted.

## Figures and Tables

**Table 1:** Characteristics of the included randomized controlled trials.

	Study	Program		Male/				
Author	Period	Timing	Grouping	Female	Age	Chemo.	Surgery	RDT
Carnaby-	2001 ~	During	1. UC	15/5	54	10	8	20
Mann	2004		2. Exercise	11/7	60	6	6	18
			3. E + I	18/2	59	6	8	20
Molen	2006 ~	Before,	1. Exercise	Total:	62.5	28	0	28
(2014)	2008	during,	2. E + I	44/11	57	27	0	27
		and after						
Høgdal	2009 ~	During and	1. UC	33/14	58.5	30	10	47
	2010	after	2. Exercise	37/13	58.6	36	8	50
Loorents	2009 ~	After	1. UC	26/7	60.2	18	14	33
	2013		2. E + I	27/6	59.3	17	16	33
Zatarain	2012 ~	During and	1. Exercise	18/2	57.7	20	6	20
	2014	after	2. E + I	18/2	57	20	7	20
Bragante	2014 ~	Before and	1. UC	26/4	58.5	18	14	30
	2015	during	2. Exercise	25/5	58.7	23	10	30
			3. E + I	25/5	54.7	20	18	30
Sandler	2016 ~	After	1. Early exercise	8/6	NR	NR	14	7
	2018		2. Late exercise	6/3	NR	NR	9	6
Carnaby	NR	During	1. UC	Overall:28	--	--	--	--
			2. Exercise	Overall:52	--	--	--	--
			3. Tutoring	Overall:50	--	--	--	--
			Overall:		59.1	99	NR	31
Lee	NR	Before and	1. E + I	24/10	NR	25	20	34
		after	2. E + I	25/12	NR	23	26	37
Wang	NR	After	1. E + I	26/4	57.7	2	30	6
			2. E + I + P (Tri-integrated)	28/2	54.27	3	30	3
Pan	NR	During &	1. E + P	31/21	51.7	NR	NR	52
		after	2. UC	30/22	52.1	NR	NR	52

E + P, exercise with phone call follow up; E + I, exercise and instrument; E + I + P, exercise and instrument and phone call follow up; NR, no report; RDT, radiotherapy; UC, usual care.

**Table 2:** Confidence rating.

Comparison	Studies	Within-Study Bias	Reporting Bias	Indirectness	Imprecision	Heterogeneity	Incoherence	Confidence Rating
Short-Term Trismus								
E + I vs. E	1	Some concerns	Unsuspected	No concerns	Major concerns	No concerns	No concerns	Low
E + I vs. E + I + P	1	No concerns	Unsuspected	No concerns	Some concerns	Some concerns	No concerns	Moderate
E + I vs. UC	2	Some concerns	Unsuspected	No concerns	Major concerns	No concerns	No concerns	Very low
E vs. UC	1	Some concerns	Unsuspected	No concerns	Major concerns	No concerns	No concerns	Very low
E + P vs. UC	1	Some concerns	Unsuspected	No concerns	Major concerns	No concerns	No concerns	Very low
E + I vs. E + P	0	Some concerns	Unsuspected	Major concerns	Major concerns	No concerns	No concerns	Very low
E vs. E + P	0	Some concerns	Unsuspected	Major concerns	Major concerns	No concerns	No concerns	Very low
E vs. E + I + P	0	Some concerns	Unsuspected	Major concerns	Major concerns	No concerns	No concerns	Very low
E + P vs. E + I + P	0	Some concerns	Unsuspected	Major concerns	Major concerns	No concerns	No concerns	Very low
E + I + P vs. UC	0	Some concerns	Unsuspected	Major concerns	Some concerns	Some concerns	No concerns	Very low
**Longer-term trismus**								
E + I vs. E	3	Some concerns	Undetected	No concerns	No concerns	Major concerns	No concerns	Low
E + I vs. UC	1	Some concerns	Undetected	No concerns	Some concerns	Some concerns	No concerns	Moderate
E vs. UC	2	Major concerns	Undetected	No concerns	Some concerns	Some concerns	No concerns	Low
E + P vs. UC	1	Some concerns	Undetected	No concerns	No concerns	No concerns	No concerns	High
E + I vs. E + P	0	Some concerns	Undetected	Major concerns	Major concerns	No concerns	No concerns	Very low
E vs. E + P	0	Some concerns	Undetected	Major concerns	No concerns	Some concerns	No concerns	Low
**Short-term mouth opening level**								
E + I vs. E	4	Some concerns	Undetected	No concerns	No concerns	Major concerns	No concerns	Low
E + I vs. E + I + P	1	No concerns	Undetected	No concerns	No concerns	No concerns	No concerns	High
E + I vs. UC	2	Some concerns	Undetected	No concerns	No concerns	Some concerns	No concerns	Moderate
E vs. UC	2	Some concerns	Undetected	No concerns	No concerns	Some concerns	No concerns	Moderate
E + P vs. UC	1	Some concerns	Undetected	No concerns	No concerns	Some concerns	No concerns	Moderate
E + I vs. E + P	0	Some concerns	Undetected	Major concerns	Some concerns	Some concerns	No concerns	Very low
E vs. E + P	0	Some concerns	Undetected	Major concerns	Some concerns	Some concerns	No concerns	Very low
E vs. E + I + P	0	Some concerns	Undetected	Major concerns	No concerns	No concerns	No concerns	Low
E + P vs. E + I + P	0	Some concerns	Undetected	Major concerns	No concerns	Some concerns	No concerns	Very low
E + I + P vs. UC	0	Some concerns	Undetected	Major concerns	No concerns	No concerns	No concerns	Low
**Longer-term mouth opening level**								
E + I vs. E	3	Some concerns	Undetected	No concerns	No concerns	Major concerns	No concerns	Low
E + I vs. UC	1	Some concerns	Undetected	No concerns	No concerns	Some concerns	No concerns	Moderate
E vs. UC	1	Some concerns	Undetected	No concerns	No concerns	Major concerns	No concerns	Low
E + P vs. UC	1	Some concerns	Undetected	No concerns	No concerns	Some concerns	No concerns	Moderate
E + I vs. E + P	0	Some concerns	Undetected	Major concerns	No concerns	Major concerns	No concerns	Very low
E vs. E + P	0	Some concerns	Undetected	Major concerns	No concerns	Major concerns	No concerns	Very low

E, exercise alone; E + P, exercise with phone call follow up; E + I, exercise and instrument; E + I + P, exercise and instrument and phone call follow up; UC, usual care.

## Supplementary Materials

The following supporting information can be downloaded at: https://www.mdpi.com/article/10.3390/healthcare10030442/s1, Material S1: Databases and search strategy, Table S2: Risk of bias assessment, Figure S3: Forest plot of direct evidence on short-term trismus rate, Figure S4: P-Score of short-term trismus rate, Figure S5: Forest plot of direct evidence on longer-term trismus rate, Figure S6: Forest plot of direct evidence on short-term mouth opening level, Figure S7: Forest plot of direct evidence on longer-term mouth opening level, Figure S8: Small-study effect in network meta-analysis of short-term trismus rate, Figure S9: Small-study effect in network meta-analysis of longer-term trismus rate, Figure S10: Small-study effect in network meta-analysis of short-term mouth opening level, Figure S11: Small-study effect in network meta-analysis of longer-term mouth opening level and Figure S12: P-curve of longer-term mouth opening level).
