# Supplementary material for: Exercise for Trismus Prevention in Patients with Head and Neck Cancer: A Network Meta-Analysis of Randomized Controlled Trials
Source: Healthcare (Basel). 2022 Feb 26;10(3):442. doi: 10.3390/healthcare10030442 (PMC8951417; doi:10.3390/healthcare10030442)
Supplement: Supplementary file 1 [file healthcare-10-00442-s001.zip › healthcare-1536921 Supplementary 220308.pdf]

Healthcare

Exercise for Trismus Prevention in Patients with Head and Neck Cancer: A Network Meta-analysis of Randomized Controlled Trials

(Supplementary)

Authors:

Ya-Hui Wang, MSc, S.T.[1,+], Yi-Ai Huang, S.T.[1,+], I-Hui Chen, Ph.D., Asso. Prof. [2,+], Wen-Hsuan Hou, MD, Ph.D., MPH, Prof.[3,4,5,6,*], Yi-No Kang, Consultant, M.A.[6,7,8,9,10,*]

Affiliations:

1. Division of Speech Therapy, Department of Physical Medicine and Rehabilitation, Wan Fang Hospital, Taipei Medical University, Taipei, Taiwan
2. School of Nursing, College of Nursing, Taipei Medical University, Taipei, Taiwan
3. Master Program in Long-Term Care, College of Nursing, Taipei Medical University, Taipei, Taiwan
4. Department of Physical Medicine and Rehabilitation, Taipei Medical University Hospital, Taipei, Taiwan
5. Center of Evidence-Based Medicine, Department of Education, Taipei Medical University Hospital, Taipei, dTaiwan
6. Cochrane Taiwan, Taipei Medical University, Taipei, Taiwan
7. Evidence-Based Medicine Center, Wan Fang Hospital, Taipei Medical University, Taipei, Taiwan
8. Research Center of Big Data and Meta-analysis, Wan Fang Hospital, Taipei Medical University, Taipei, Taiwan
9. Institute of Health Policy & Management, College of Public Health, National Taiwan University, Taipei, Taiwan
10. Department of Health Care Management, College of Health Technology, National Taipei University of Nursing Health Sciences, Taipei, Taiwan

\+ Co-first author: Ya-Hui Wang, MSc, S.T., Yi-Ai Huang, S.T., and Hui Chen Ph.D., Prof. contributed equally.

* Correspondence: Correspondence: Wen-Hsuan Hou, MD, Ph.D., MPH, Prof.Master Program in Long-Term Care, College of Nursing, Taipei Medical University No. 250, Wuxing Street, Xinyi District, Taipei 11031, Taiwan @tmu.edu.twYi-No Kang, M.A., consultant Evidence-Based Medicine Center, Wan Fang Hospital, Taipei Medical University No. 111, Section 3, Xinglong Road, Taipei 11696, Taiwan

Supplementary

Supplementary Material S1. Databases and search strategy

Supplementary Table S2. Risk of bias assessment

Supplementary Figure S3. Forest plot of direct evidence on short-term trismus rate

Supplementary Figure S4. P-Score of short-term trismus rate

Supplementary Figure S5. Forest plot of direct evidence on longer-term trismus rate

Supplementary Figure S6. Forest plot of direct evidence on short-term mouth opening level

Supplementary Figure S7. Forest plot of direct evidence on longer-term mouth opening level

Supplementary Figure S8. Small-study effect in network meta-analysis of short-term trismus rate

Supplementary Figure S9. Small-study effect in network meta-analysis of longer-term trismus rate

Supplementary Figure S10. Small-study effect in network meta-analysis of short-term mouth opening level

Supplementary Figure S11. Small-study effect in network meta-analysis of longer-term mouth opening level

Supplementary Figure S12. P-curve of longer-term mouth opening level

Supplementary Material S1
Search strategy (Primary search strategy)

Primary search strategy

#1. trismus OR OR OR) AND (OR OR OR OR) AND (OR OR
#2. lockjaw
#3. mouth opening
#4. jaw opening
#5. #1 OR #2 OR #3 OR #4
#6. cancer
#7. carcinoma
#8. tumor
#9. head and neck cancer
#10. head and neck tumor
#11. #6 OR #7 OR #8 OR #9 OR #10
#12. prevent
#13. prevention
#14. prevent*
#15. #12 OR #13 OR #14
#16. #5 AND #11 AND #15

Example 1
Syntax in Cochrane Library (including CENTRAL):

(trismus OR lockjaw OR mouth opening OR jaw opening) AND (cancer OR carcinoma OR tumor OR head and neck cancer OR head and neck tumor) AND (prevent OR prevention OR prevent*)

Example 2
Search details in PUBMED :

("trismus"[MeSH Terms] OR "trismus"[All Fields] OR ("trismus"[MeSH Terms] OR "trismus"[All Fields] OR "lockjaw"[All Fields]) OR (("mouth"[MeSH Terms] OR "mouth"[All Fields] OR "mouths"[All Fields] OR "mouth s"[All Fields] OR "mouthed"[All Fields] OR "mouthful"[All Fields] OR "mouthfuls"[All Fields] OR "mouthing"[All Fields]) AND ("opened"[All Fields] OR "opening"[All Fields] OR "openings"[All Fields] OR "opens"[All Fields])) OR (("jaw"[MeSH Terms] OR "jaw"[All Fields]) AND ("opened"[All Fields] OR "opening"[All Fields] OR "openings"[All Fields] OR "opens"[All Fields]))) AND ("cancer s"[All Fields] OR "cancerated"[All Fields] OR "canceration"[All Fields] OR "cancerization"[All Fields] OR "cancerized"[All Fields] OR "cancerous"[All Fields] OR "neoplasms"[MeSH Terms] OR "neoplasms"[All Fields] OR "cancer"[All Fields] OR "cancers"[All Fields] OR ("carcinoma"[MeSH Terms] OR "carcinoma"[All Fields] OR "carcinomas"[All Fields] OR "carcinoma s"[All Fields]) OR ("cysts"[MeSH Terms] OR "cysts"[All Fields] OR "cyst"[All Fields] OR "neurofibroma"[MeSH Terms] OR "neurofibroma"[All Fields] OR "neurofibromas"[All Fields] OR "tumor s"[All Fields] OR "tumoral"[All Fields] OR "tumorous"[All Fields] OR "tumour"[All Fields] OR "neoplasms"[MeSH Terms] OR "neoplasms"[All Fields] OR "tumor"[All Fields] OR "tumour s"[All Fields] OR "tumoural"[All Fields] OR "tumourous"[All Fields] OR "tumours"[All Fields] OR "tumors"[All Fields]) OR ("head and neck neoplasms"[MeSH Terms] OR ("head"[All Fields] AND "neck"[All Fields] AND "neoplasms"[All Fields]) OR "head and neck neoplasms"[All Fields] OR ("head"[All Fields] AND "neck"[All Fields] AND "cancer"[All Fields]) OR "head and neck cancer"[All Fields]) OR ("head and neck neoplasms"[MeSH Terms] OR ("head"[All Fields] AND "neck"[All Fields] AND "neoplasms"[All Fields]) OR "head and neck neoplasms"[All Fields] OR ("head"[All Fields] AND "neck"[All Fields] AND "tumor"[All Fields]) OR "head and neck tumor"[All Fields])) AND ("prevent"[All Fields] OR "preventability"[All Fields] OR "preventable"[All Fields] OR "preventative"[All Fields] OR "preventatively"[All Fields] OR "preventatives"[All Fields] OR "prevented"[All Fields] OR "preventing"[All Fields] OR "prevention and control"[MeSH Subheading] OR ("prevention"[All Fields] AND "control"[All Fields]) OR "prevention and control"[All Fields] OR "prevention"[All Fields] OR "prevention s"[All Fields] OR "preventions"[All Fields] OR "preventive"[All Fields] OR "preventively"[All Fields] OR "preventives"[All Fields] OR "prevents"[All Fields] OR ("prevent"[All Fields] OR "preventability"[All Fields] OR "preventable"[All Fields] OR "preventative"[All Fields] OR "preventatively"[All Fields] OR "preventatives"[All Fields] OR "prevented"[All Fields] OR "preventing"[All Fields] OR "prevention and control"[MeSH Subheading] OR ("prevention"[All Fields] AND "control"[All Fields]) OR "prevention and control"[All Fields] OR "prevention"[All Fields] OR "prevention s"[All Fields] OR "preventions"[All Fields] OR "preventive"[All Fields] OR "preventively"[All Fields] OR "preventives"[All Fields] OR "prevents"[All Fields]) OR "prevent*"[All Fields])

Supplementary Table S2

Risk of bias

Item	Bragante	Carnaby	Carnaby-Mann	Høgdal	Lee	Loorents	Van Der Molen	Wang	Zatarain	Sandler	Pan
Relevant information											
Randomization	Computer generated	No report (by CRT status)	Computer generated	Computer generated	Minimisation method	Computer generated	Computer generated	Computer generated	Computer generated	computer-generated	Random table
Concealment	sealed, opaque, brown envelopes	No report	Computer generated	according to operation (Y/N)	No report	Computer generated	Computer generated	Opaque, sealed envelopes.	Computer generated	computer-generated	No Report
Blinding	Analysist	No blind	Assessor	Assessor	No Report	No Report	No Report	Patients, personnel, assessor	No Report	Surgeons (referral)	No Report
Follow-up duration	12 months	3 months	6 months	12 months	6 months	12 months	24 months	12 weeks after discharged	6 months	6 months	24 months
Intervention duration	1 day before ~ end of RDT	6 weeks	During CRT	5 – 6 weeks	6months	12 months	12 months	12-week	3 months	1 session	12 months
Intensity	4 times/day +weekly supervised	No report	2 times/day	5 times/day	5 sessions/day	5 times/day	3times/day	12-week	3times/day	3times/day	No Report
Loss follow-up	13/90(14.4%)	29/130(22%)	24/58(41.4%)[a]	24/100 (24%)	30/71 (42.3%)	24/66(36.4%)	26/55 (47%)	8/60 (12%)	7/40 (18%)	6/30 (20%)[a]	No Report
Analysis type	ITT	PP	PP	PP	PP	PP	PP	PP	PP	PP	ITT
Sponsor	Sponsored [b]	No	No	Sponsored [c]	No	Sponsored [d]	Sponsored [e]	Sponsored [f]	Sponsored[g]	Sponsored[h]	No

a: mortality was excluded. b: Funding agency, NoCoordena¸c~ao de Aperfei¸coamento de Pessoal de Nıvel Superior, Brazil (CAPES) (Finance Code 001). c: The Association of Danish Physiotherapists Research Fund, The Frantz Hoffmann Memorial Fund, The Capital Region of Denmark, The Copenhagen Trial Unit, and The Research-Initiative Fund of the Copenhagen University Hospital. d; The Swedish Cancer Society, the Medical Research Council of Southeast Sweden, the Department of Radiation Oncology at the University Hospital in Linköping, the County Hospital in Jönköping, the Department of Medical and Health Sciences, division of Nursing Science, Linköping University, and the County Council of Östergötland. e: Atos Medical, Ho¨rby, Sweden. f: Ministry of Science and Technology, Taiwan. g: Dynasplint Sytems, Inc, provided all Jaw Dynasplint Systems for the clinical trial. h: Mount Sinai Health System; Icahn School of Medicine. CRT, chemoradiotherapy; ITT, intention to treat; PP, per-protocol; RDT, radiation therapy.

Item	Bragante [a]	Carnaby	Carnaby-Mann	Høgdal [b]	Lee	Loorents [c]	Van Der Molen	Wang	Zatarain	Sandler	Pan
Randomization	Low	Unclear	Low	Low	High	Low	Low	Low	Low	Low	Low
Concealment	Low	Unclear	Low	Low	Unclear	Low	Low	Low	Low	Low	Unclear
Performance	High	High	High	High	High	High	High	Low	High	High	High
Detection	High	High	Low	Low	High	High	High	Low	High	High	High
Attrition	Low	High	High	High	High	High	High	Unclear	Unclear	High	Low
Reporting	Unclear	High	Unclear	Unclear	Unclear	Low	Unclear	Unclear	Unclear	Unclear	Unclear
Other source	Unclear	Low	Low	Unclear	Low	Unclear	High	Unclear	High	Unclear	Unclear

a: ClinicalTrials.gov (No. NCT02094690), and at the Brazilian Clinical Trials Registry (No. RBR89 mdvw). b: ClinicalTrials.gov (No. NCT00780312). c: ClinicalTrials.gov (No. NCT01354548 NCT01354548).

Supplementary Figure S3

Forest plot of direct evidence on short-term trismus rate

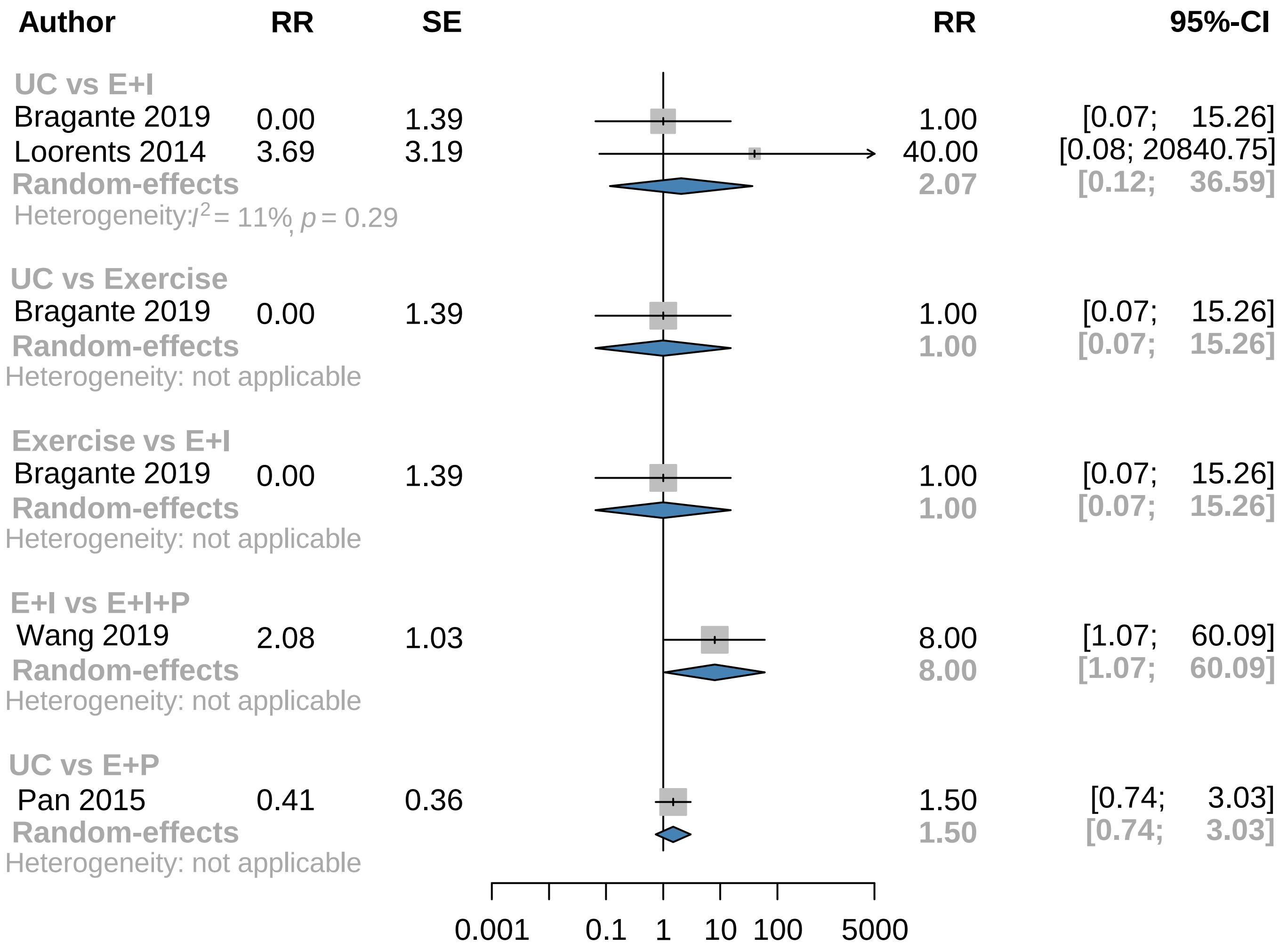

Supplementary Figure S4

P-Score of short-term trismus rate

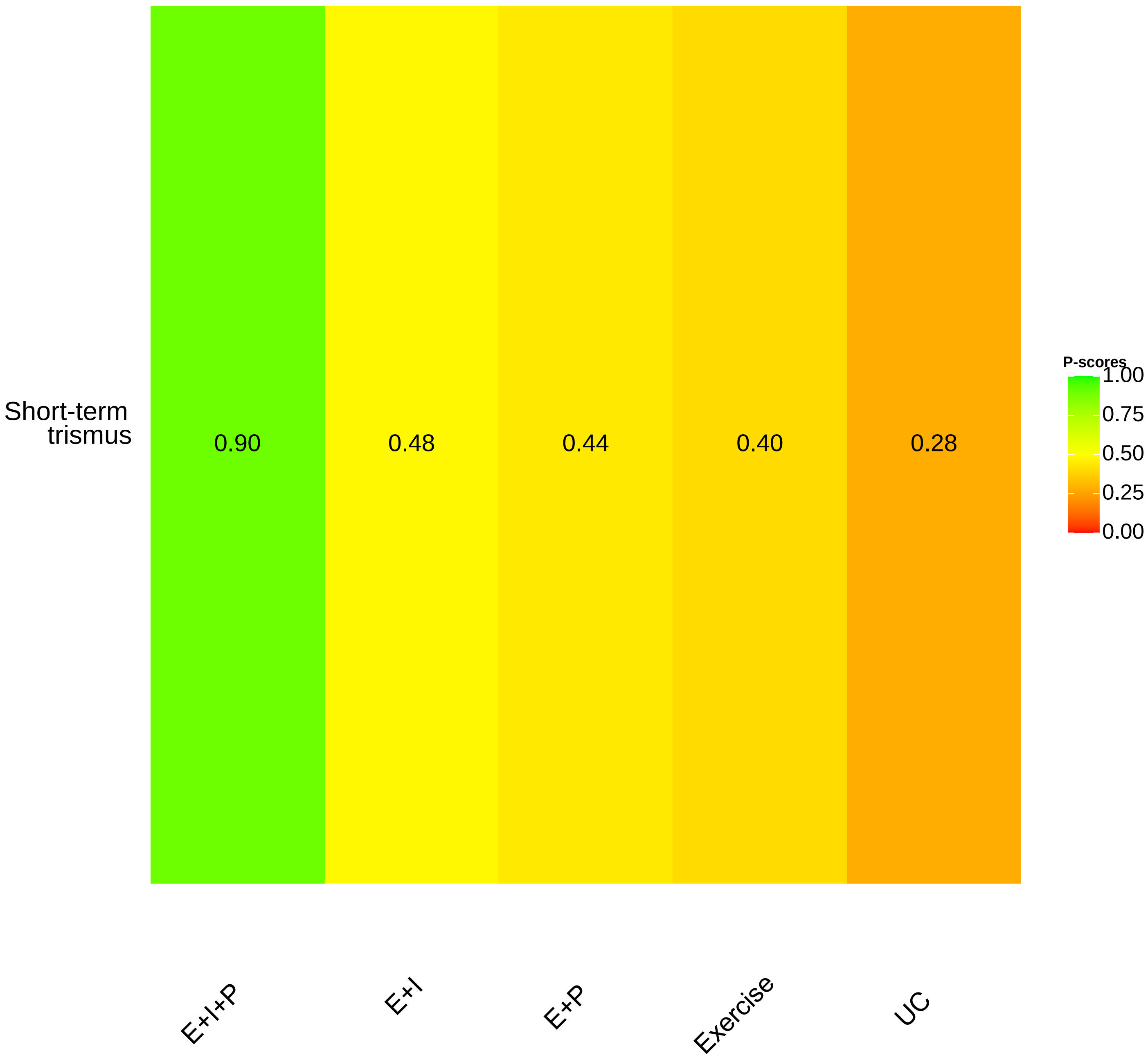

Supplementary Figure S5

Forest plot of direct evidence on longer-term trismus rate

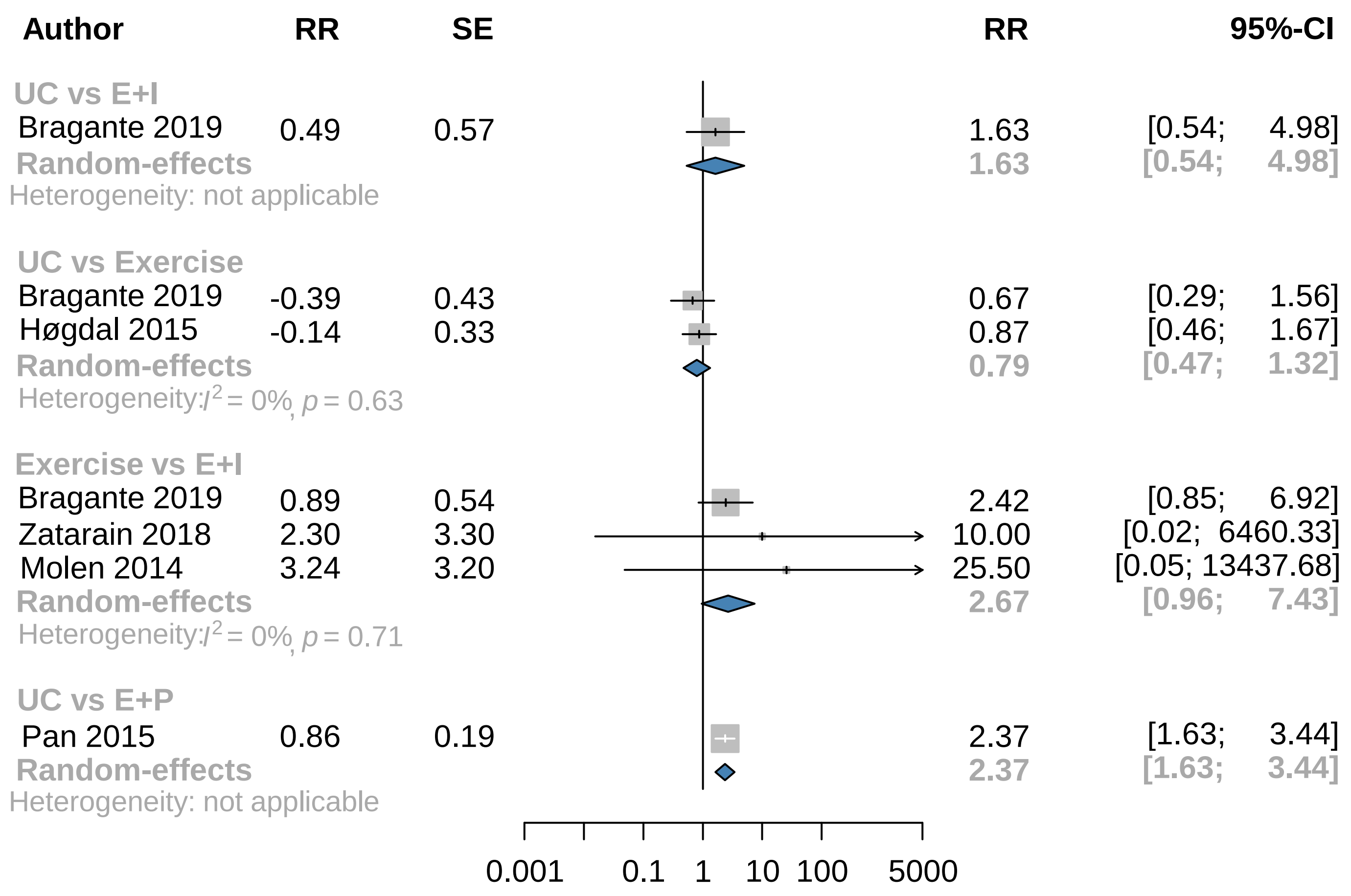

Supplementary Figure S6

Forest plot of direct evidence on short-term mouth opening level

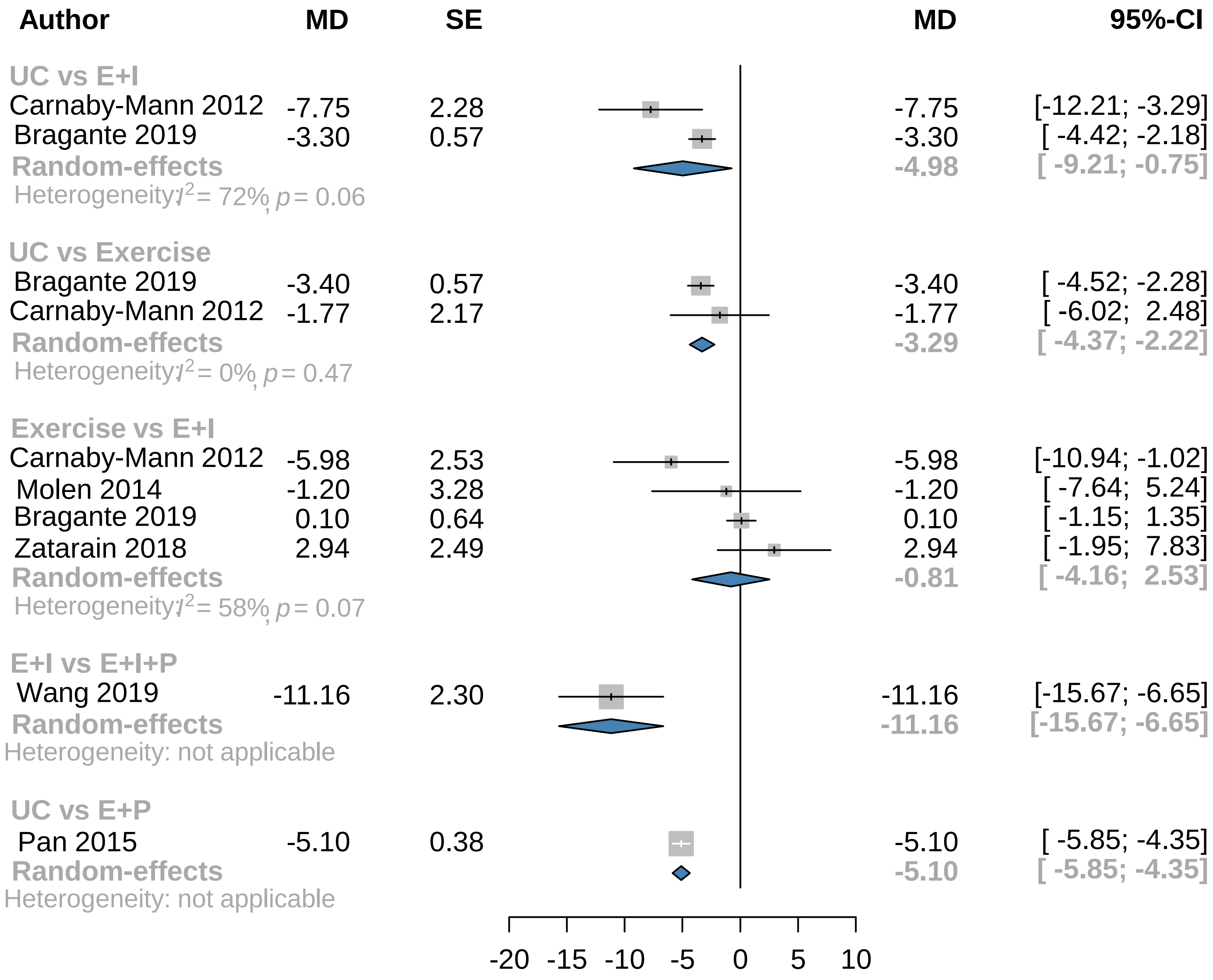

Supplementary Figure S7

Forest plot of direct evidence on longer-term mouth opening level

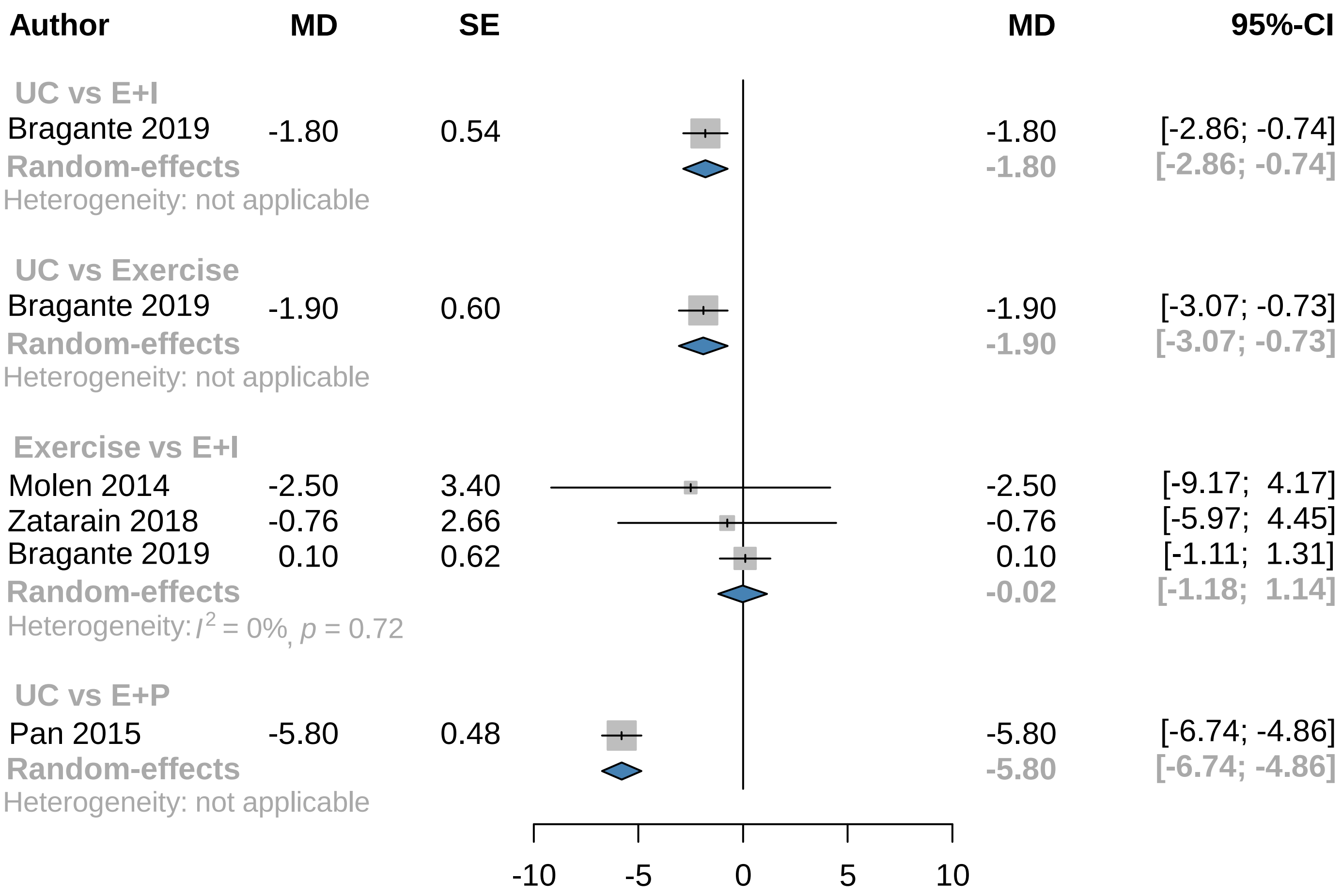

Supplementary Figure S8

Small-study effect in network meta-analysis of short-term trismus rate

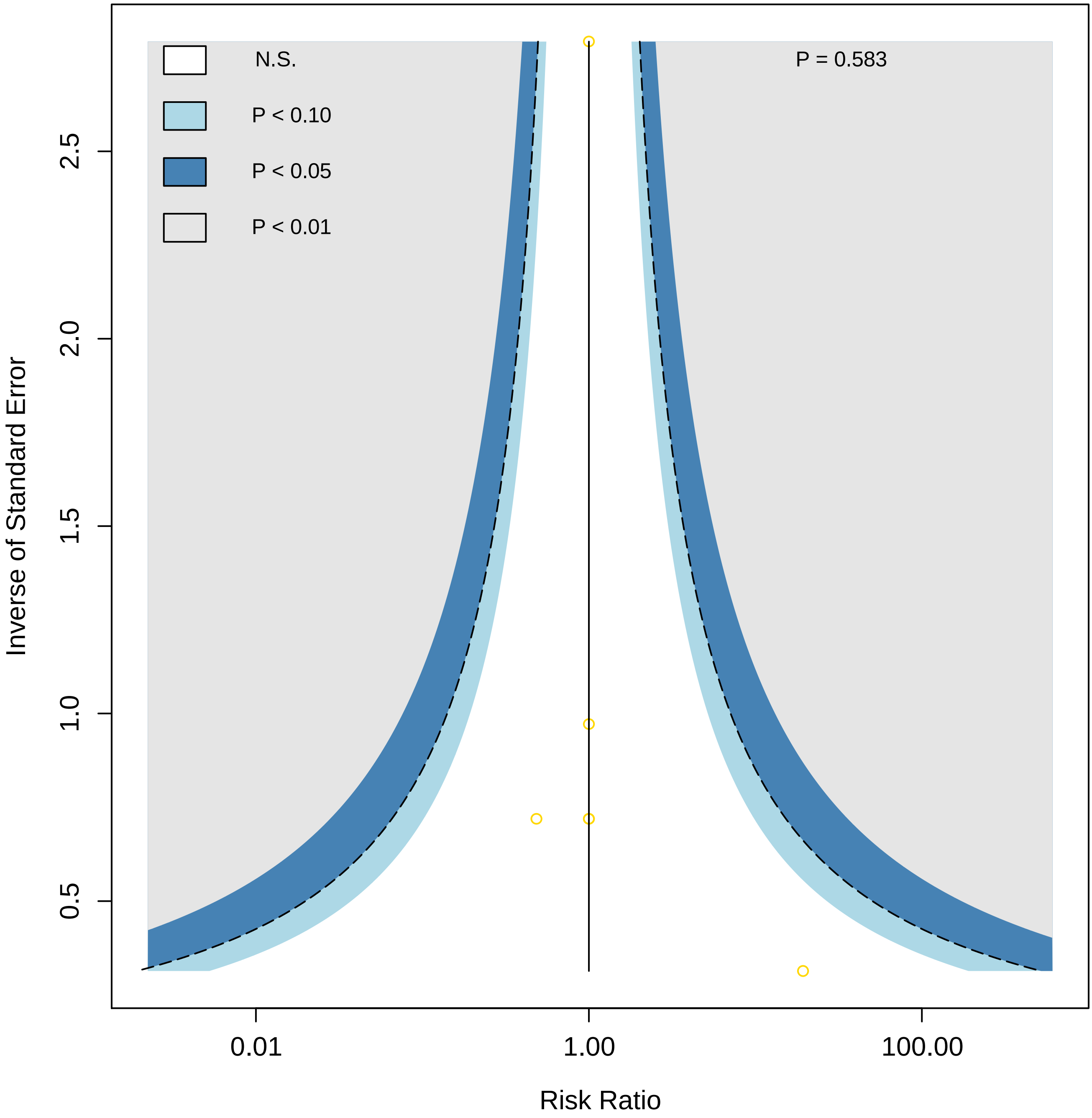

Supplementary Figure S9

Small-study effect in network meta-analysis of longer-term trismus rate

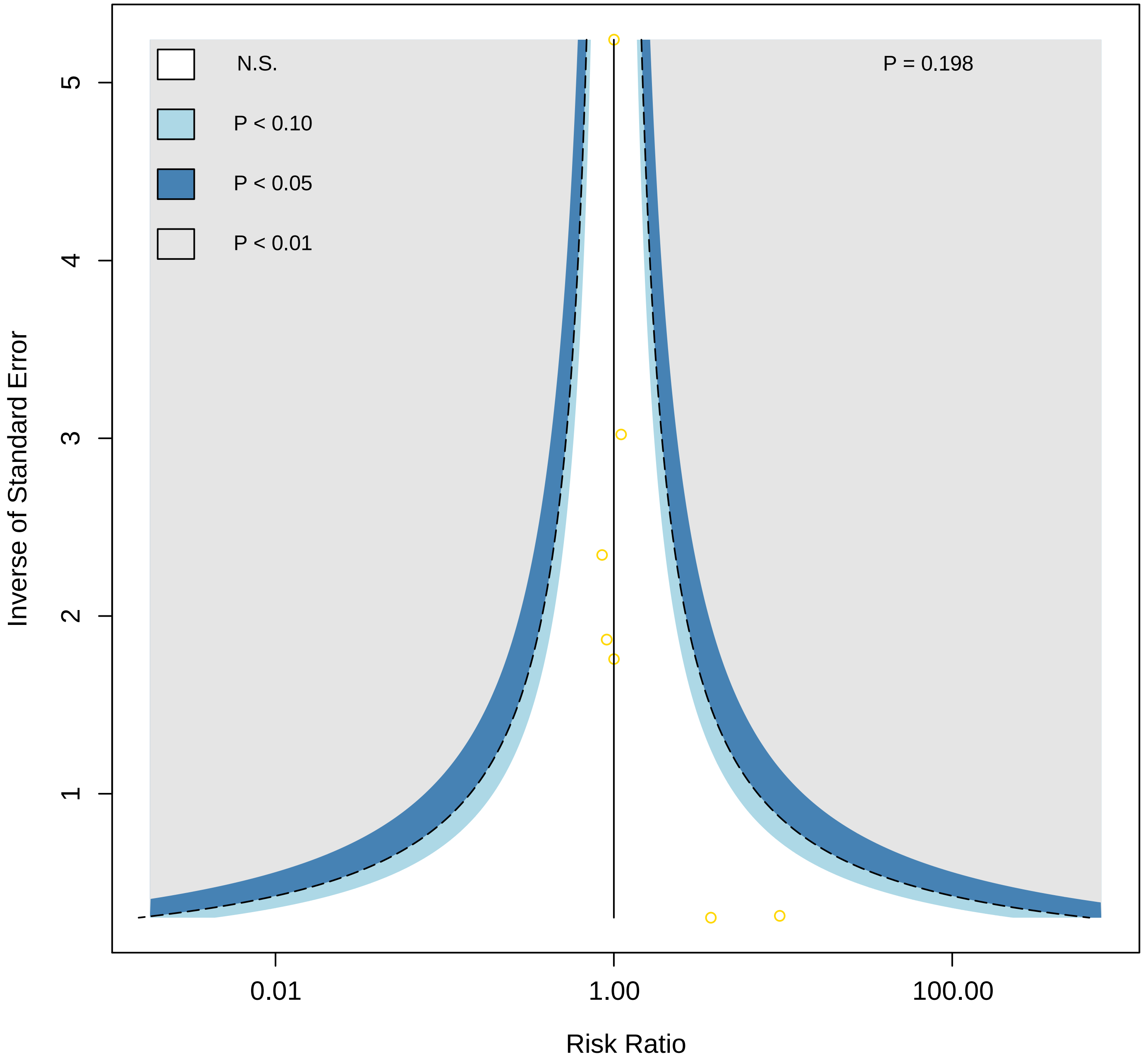

Supplementary Figure S10

Small-study effect in network meta-analysis of short-term mouth opening level

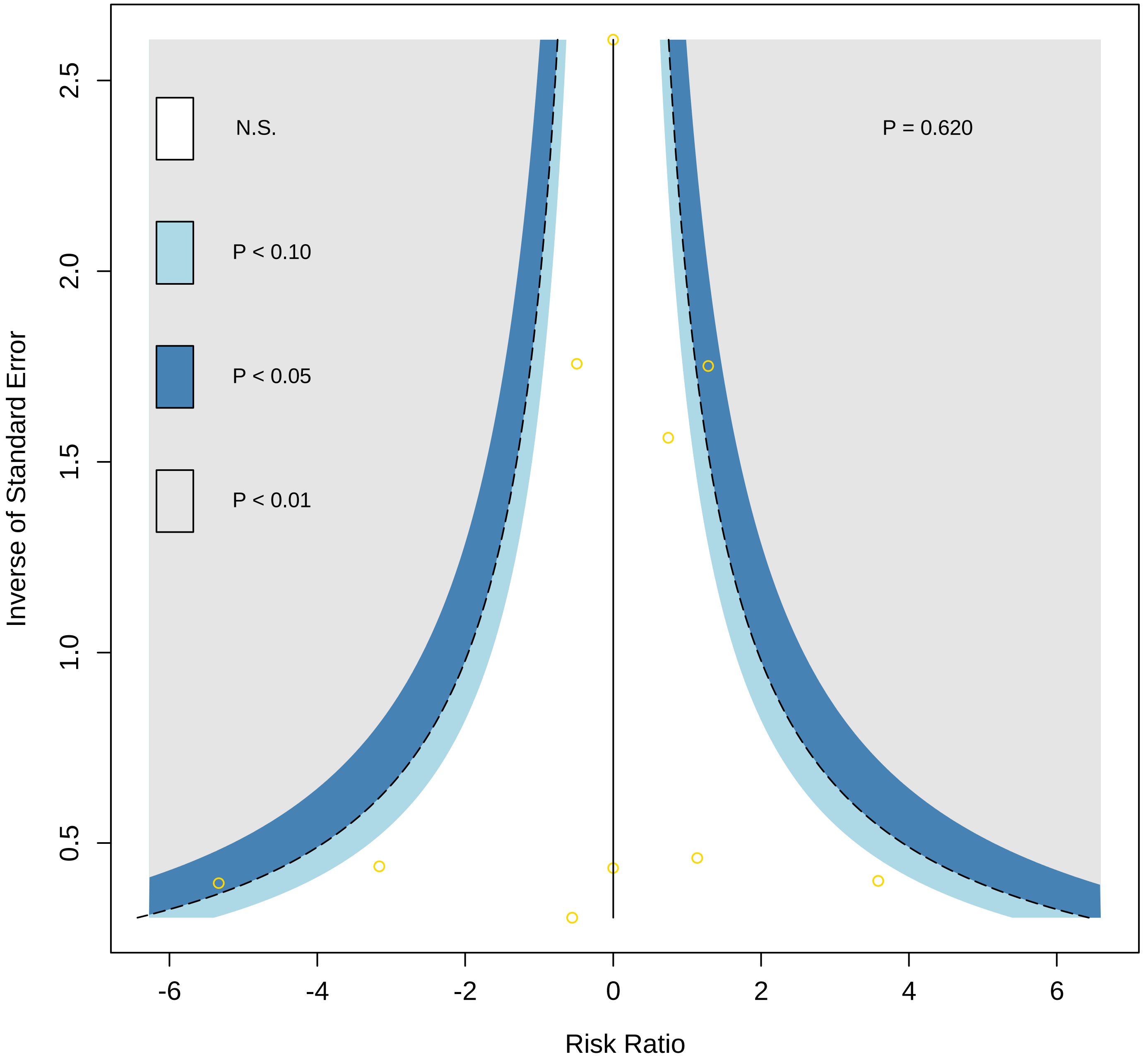

Supplementary Figure S11

Small-study effect in network meta-analysis of longer-term mouth opening level

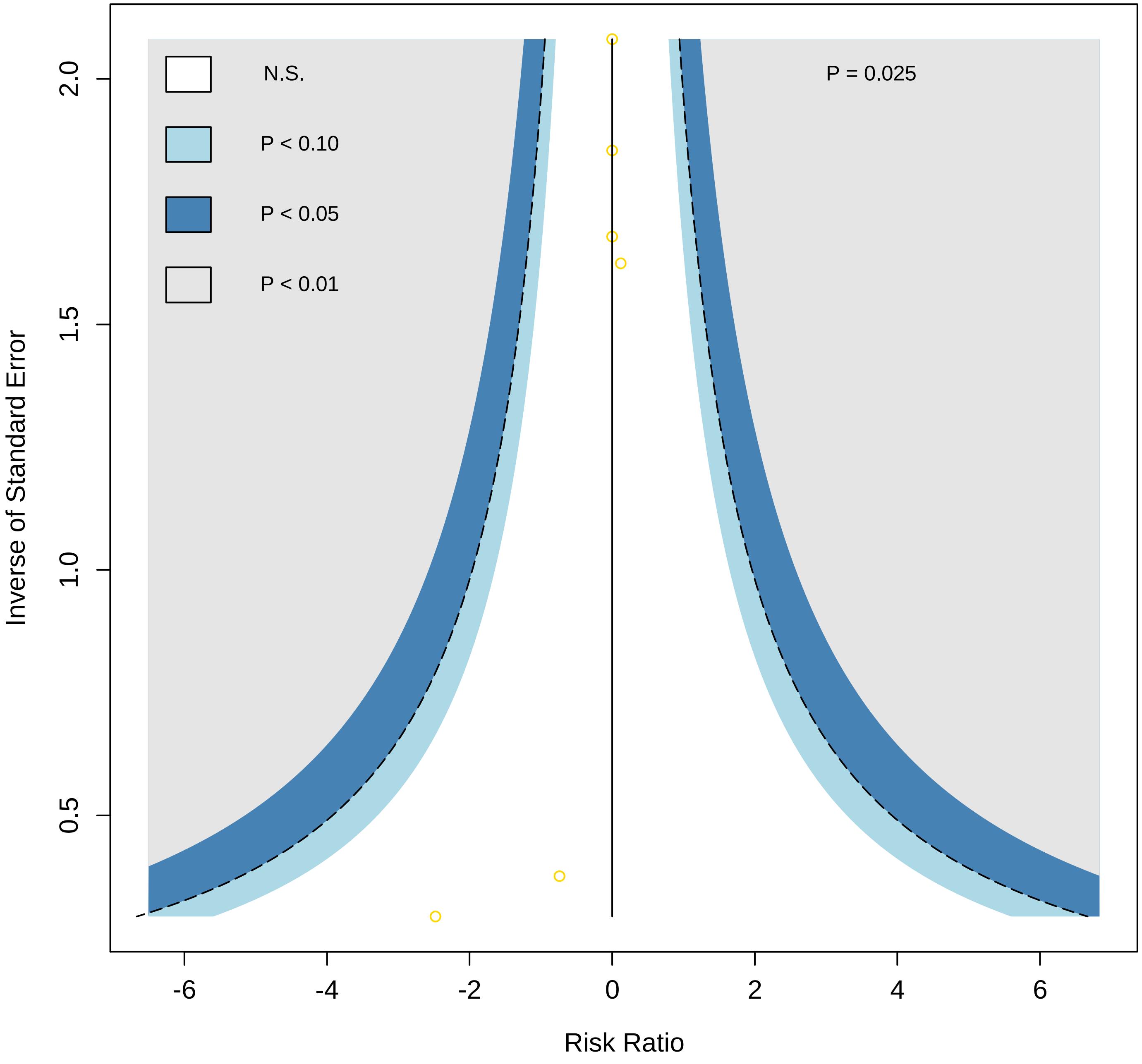

Supplementary Figure S12

P-curve of longer-term mouth opening level

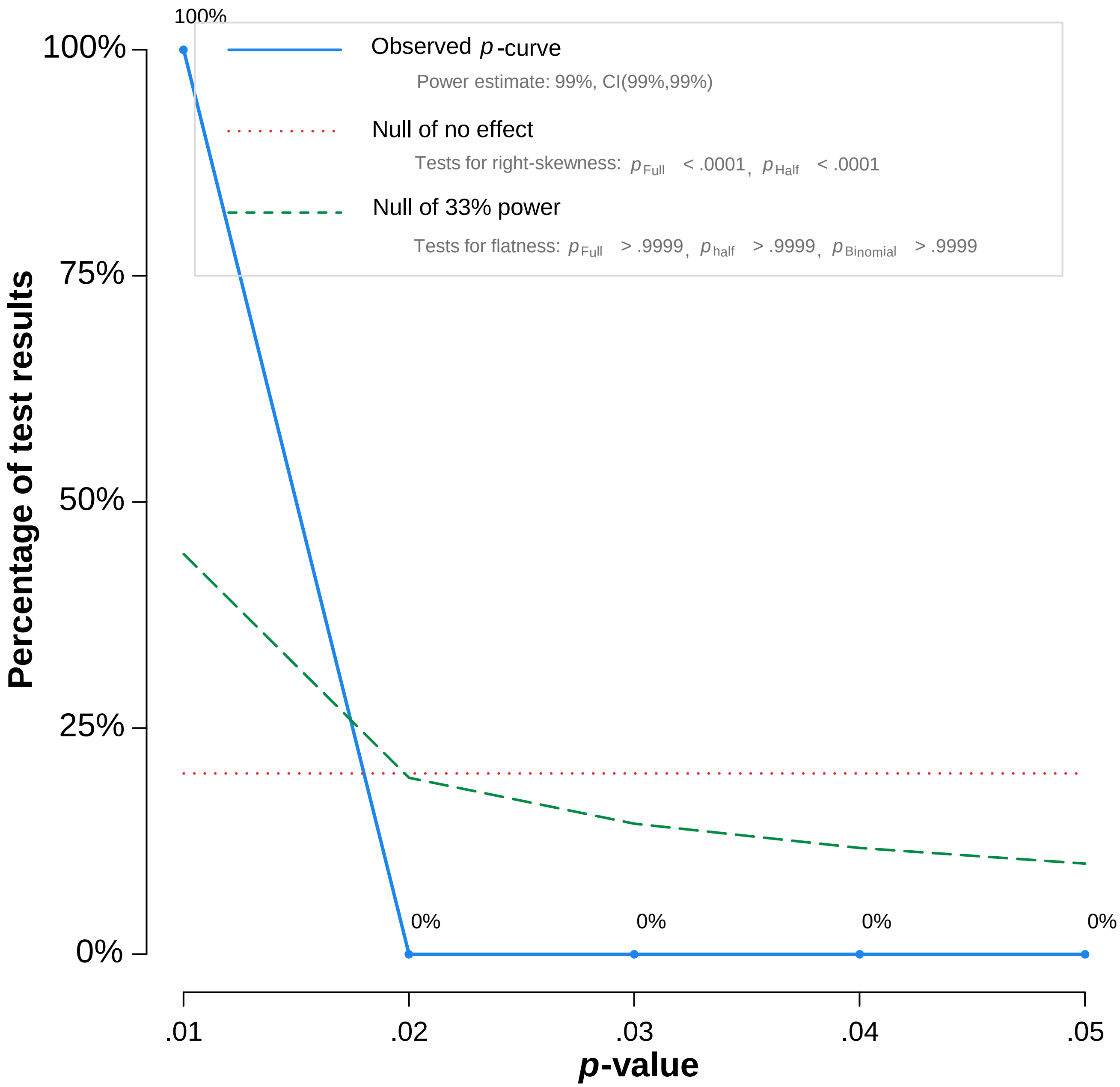

Note: The observed *p*-curve includes 3 statistically significant ($p < .05$) results, of which 3 are $p < .025$. There were 3 additional results entered but excluded from *p*-curve because they were $p > .05$.
